# Multiple Dental Inclusion in Monozygotic Twins with Congenital Visual Impairment

**DOI:** 10.1155/2020/8856206

**Published:** 2020-08-06

**Authors:** G. Galluccio, A. Impellizzeri, A. A. De Stefano, E. Serritella, E. Guercio Monaco

**Affiliations:** ^1^Department of Oral and Maxillo Facial Sciences, Faculty of Medicine and Dentistry, Sapienza Università di Roma, Italy; ^2^Department of Orthodontics, Faculty of Dentistry, University Central of Venezuela, Caracas, Venezuela

## Abstract

The study presents two monozygotic twins (MZ) with multiple impacted teeth, affecting the upper canines and lower second molars, as well as congenital aniridia. The clinical aspect of the upper canines is peculiar because of the different positions—palatal in one and buccal in the other twin. Studies reporting different scenarios of impaction in monozygotic twins can contribute more data to the debate on tooth eruption aetiology and more so in this case because of the association with a genetic panocular disease. *Patients' Concerns*. The patients were referred by a general dentist, who diagnosed the presence of multiple inclusions. *Diagnostic Study*. Both patients showed severe malocclusion, classified as grade 5 of the Index of Orthodontic Treatment Need (IOTN). The MZ showed class I malocclusion, upper and lower crowding, and impacted lower right and left second molars. A Dentascan was prescribed for the canine impaction. The impaction of the upper canine was palatal of 2.3 in one of the MZ and buccal of 1.3 in the other one. The same altered pattern of eruption of the lower second molars was identified in both twins. The proposed treatment plan contemplated orthodontic surgical recovery of the impacted elements, followed by orthodontic treatment with multibracket appliance after the extraction of the first four premolars, given the crowding entity. The use of a retraction spring action was chosen for the recovery of the lower second molars. Many aspects of the possible genetic aetiology of tooth impaction are still under discussion. The study of diseases in twins offers decisive information. Finally, the possibility that alterations in the eruptive pattern of the dental elements may be associated with other congenital problems broadens the range of investigations related to the possible aetiological causes of the inclusions in humans.

## 1. Introduction

Dental anomalies such as hypodontia, supernumerary teeth, enamel defects, and abnormalities of eruption and position are prominent in a large segment of the population.

Although treatment strategies for these conditions are steadily improving, much remains to be understood about their aetiology and pathophysiology [1].

Studies on monozygotic twins may elucidate the genetics underlying the specific aspects of malocclusions and tooth anomalies in the analysis, improving the possibilities of performing a correct treatment plane [2–7].

The presence of concordant or nonconcordant malocclusion traits in monozygotic twins is a special occasion to test the hypothesis in the aetiology of inclusions, which is still nonconcordant in the literature [6, 8].

Special interest is present when specific occlusal alterations are associated with other systemic anomalies and overall when the association is with congenital anomalies.

The case presented, of the monozygotic twins V. P. and L. P., is the concurrent presence of multiple-tooth impaction affecting the upper canines and lower second molars and congenital aniridia. This particular congenital panocular disease is described as the partial or complete absence of the iris and often recognized in association with other diseases [9]. The aetiology of aniridia is recognized as genetic; for example, it was recently identified to be referred to as a deletion in the regulatory region of PAX6 [10, 11].

### 1.1. Clinical Findings

The twins came for an orthodontic evaluation accompanied by their parents. The patients (Caucasian, 14 yo) were referred by the general dentistry after their first visit during which the observation of a panorex revealed the presence of an altered pattern of eruption on several dental elements and multiple inclusions. Both the twins had a severe visual impairment with a limitation up to shadow perception due to the presence of congenital aniridia.

The principal concern of the family was related to the misalignment of the teeth and related difficulties in maintaining proper oral hygiene. The parents and the general dentistry considered visual impairment a possible limitation in the compliance, eventually needing restorative dentistry procedures.

From the age of 6 yo, the patients had undergone regular dentistry controls and periodic procedures of oral hygiene with professional deplaquing and instructional reinforcement. The provision of further preventive procedures such as sealants or varnish was not referred.

### 1.2. Medical History

The parents reported a course of pregnancy without major problems, birth at term by a caesarean section for malposition of the foetuses, and an Apgar score of 8–10. The lactation was mixed (natural/artificial).

The twins were born from the only pregnancy of the couple, and no other sibling was known.

For both the patients, the presence of congenital aniridia was definitively diagnosed at three months of age.

The two patients showed regular somatic development (L. P.'s menarche at the age of 12.3; V. P.'s menarche at the age of 11.9).

The patients had no need for drug treatments for specific conditions.

### 1.3. Family History

Both parents appeared to be in good health. The anamnesis was negative for the presence of malocclusion and dental impaction in the family.

In medical dental history, the mother reported the need for restorations while the father reported a previous need for restorative and prosthetic dental procedures. Neither had undergone orthodontic treatment.

### 1.4. Lifestyle and Psychosocial Information

Due to their visual impairment, social interactions, as were for performing physical activity, were limited as much as possible. The schooling was regular as per the age, albeit with the need for devices and supplementary support from teachers due to the visual limitation.

The two sisters showed a strong relationship, evident in their need for each other's presence during the diagnostic and therapeutic procedures.

### 1.5. Genetic Information

Genetic assessment of the patients was not performed at birth and was refused by the parents after it because it was referred to as insignificant in the medical treatment choices of the disease and for economic reasons.

#### 1.5.1. Case # 1: Twin V. P.

The medical history of the patient reported that she was diagnosed with bilateral aniridia with congenital bilateral polar cataracts, highlighted at the age of three months, later surgically treated by removing it and by placing cosmetic intraocular implants in both eyes.

Visus was equal to 2/30 in the right eye and 1/10 in the left eye, without any possibility of improvement.

Any kind of dental or orthodontic treatment was previously performed.

**Figure 1:**
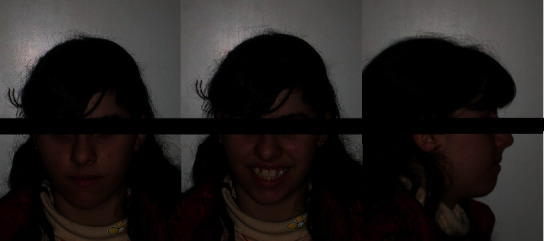
MZ V. P.'s extraoral examination.

*(1) Physical Examination*. The extraoral examination showed an increased lower facial height (dolichofacial), slight convex profile, lines of symmetry respected, and lip incompetence at rest (Figure 1).

The intraoral examination presented that the patient had late mixed dentition; it was noticeable in the presence of the arches of deciduous teeth, not consistent with the age: upper left canine and lower left second molar. Both lower second permanent molars showed a semi-inclusion with only the distal cusps visible in the mouth (Table 1).

**Figure 2:**
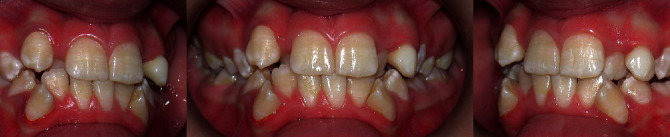
MZ V. P.'s intraoral examination.

The occlusal molar relationships were of class I on the right and the left side, while the canine relationship was undetectable because of the ectopic position of the upper right canine and the absence in the arch of the left one. There was deviation of the lower midline (Figure 2).

Overjet and overbite were increased; a severe dentoalveolar discrepancy, with evident upper and lower crowding, was evident. The oral hygiene was poor, with bacterial plaque, probably due to the limited visual capacity. Decays were present on elements 3.6 and 3.7. An amalgam restoration was present on the vestibular surface of 4.6.

*(2) Radiographic Examination*. The orthopantomography of the dental arches reveals the presence of all permanent teeth, with the upper left canine impacted; mesial inclination of the lower second molars is seen, with the mesial cusps impacted under the crown of the first molars.

**Figure 3:**
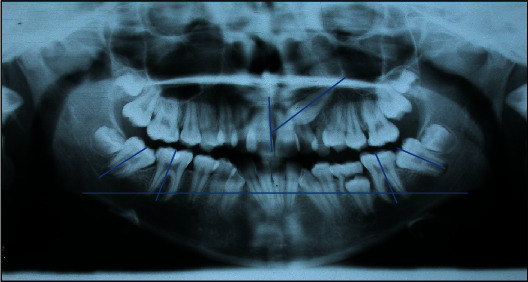
MZ V. P.'s panorex. The upper left canine *α* angle is 57°, and the vertical distance from the occlusal plane is 9.5 mm. The inclination of the second molar axis is 38° for 4.7 and 40° for 3.7.

The inclination of the second molar axis related to the first molar axis was analysed for the prognostic value of this measurement [12] and was 38° for 4.7 and 40° for 3.7 (Figure 3).

The inclination of the upper left canine was 57° as evaluated with the *α* angle, and the vertical distance from the occlusal plane was 9.5 mm. The evaluation of the alpha angle and the D-distance has a prognostic value in relation to the success of surgical orthodontic treatment. The angle is formed from the canine axis and the midline between the central incisors. The D-distance is measured from the canine cusp to the occlusal plane [13]. The evaluation of these parameters can be also read, in an early stage of dentition, as an indication of progressive canine impaction. However, these radiographic variables take on a diagnostic and prognostic significance of canine dislocation at a later age group, i.e., only after 10–11 years [14].

The presence of severe eruptive disturbances suggested the prescription of further radiographic evaluation with a TC Dentascan of the upper and lower arch.

**Figure 4:**
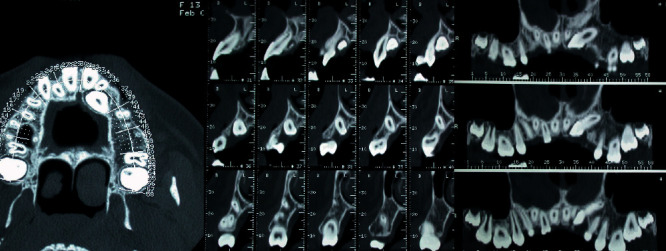
MZ V. P.'s upper arch Dentascan: upper left canine palatal impaction.

The observation revealed an inclusion of 2.3 in the palatal position. A tight contact was evident between the palatal surface of the central and lateral left incisors' roots and the crown of the impacted canine. However, no signs of root resorption were evident for the incisors. An eruptive delay of 1.4, 1.5, and 4.5 was detectable with altered axial inclination (Figure 4).

**Figure 5:**
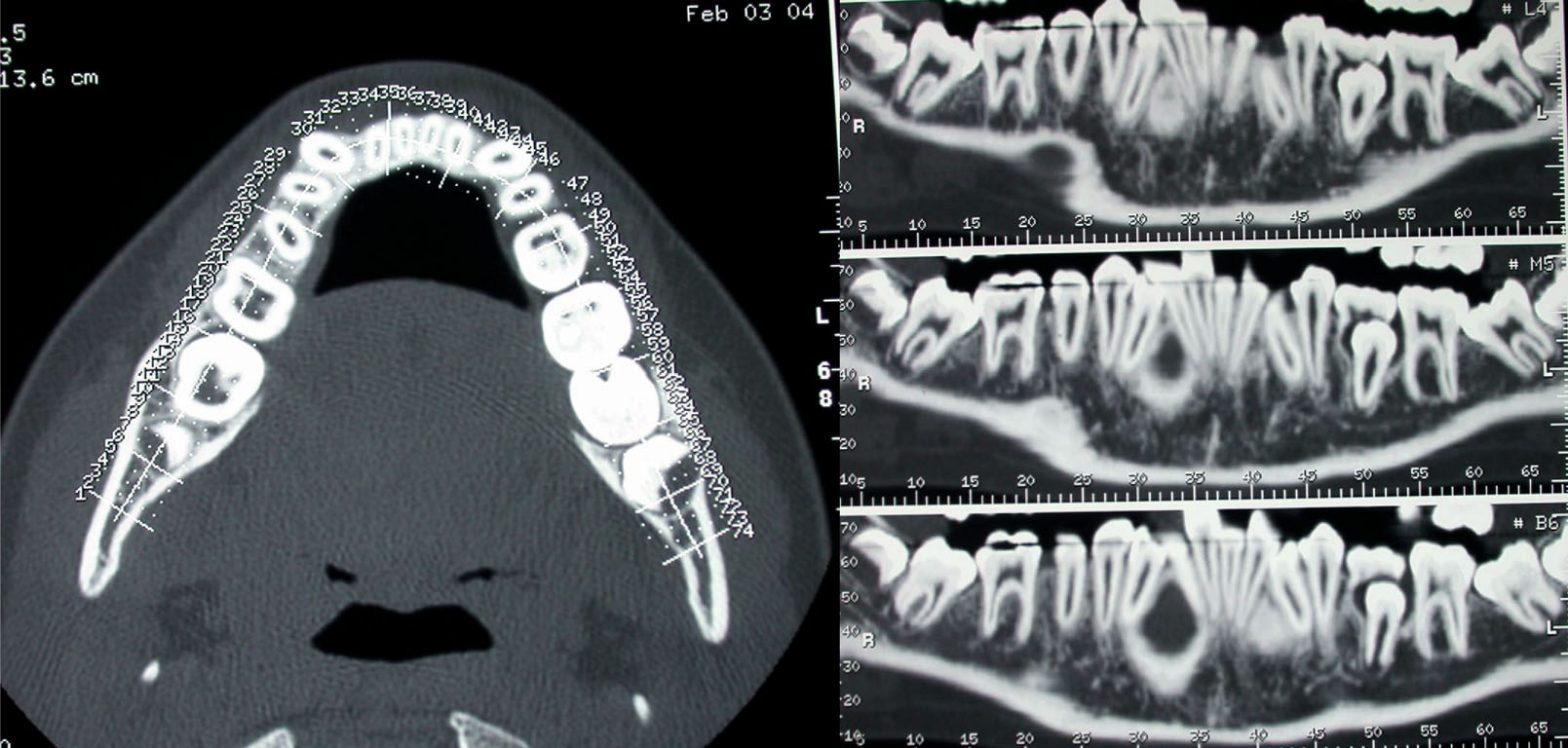
MZ V. P.'s lower arch Dentascan: mesial impaction of both the lower second molars.

The evaluation of the two lower molars showed an H stage of maturity (Demirjian dental age assessment) [15], acceptable for the age of the patient; the vestibulooral position of both the molars was centred with limited contact with the lingual cortical bone for 3.7 (Figure 5).

For both the second molars, the mesial cusps were positioned under the crown equator of the respective molars.

The cephalometric analysis of the latero-lateral X-ray is summarised in Table 2. The results show a class II malocclusion, an increase in the vertical dimension of the lower third, and the vestibularisation of the upper incisors.

*(3) Cast Analysis*. A severe dentoalveolar discrepancy was present in the upper arch, equal to −13, and the lower arch, equal to −14 (Table 1).

#### 1.5.2. Case # 2: Twin L. P.

The medical history of the patient reports that at the age of three months, she was diagnosed with bilateral aniridia with congenital bilateral polar cataracts, which was treated surgically by removing it and by placing cosmetic intraocular implants to both eyes. Secondary retinal detachment resulted in corneal opacity in the right eye.

Visus was equal to light perception in the right eye and 2/30 in the left eye, without any possibility of improvement.

Any kind of dental or orthodontic treatment was previously performed.

**Figure 6:**
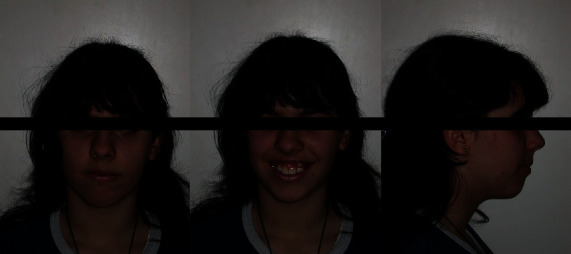
MZ L. P.'s extraoral examination.

*(1) Physical Examination*. The extraoral examination showed an increased lower facial height (dolichofacial), slight convex profile, lines of symmetry respected, and lip incompetence at rest (Figure 6).

The intraoral examination showed that the patient was in a late mixed dentition stage. The relative formula is reported in Table 3. It was noticeable in the presence of arches in deciduous teeth: upper left canine and upper and lower left second molars. This was not consistent with the age, and both lower second permanent molars showed only the distal cusps visible in the mouth.

**Figure 7:**
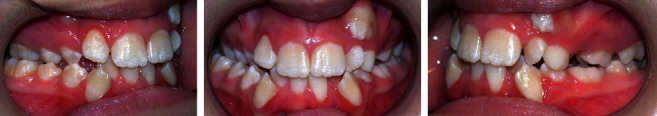
MZ L. P.'s intraoral examination.

The occlusal molar relationships were of class I on the right and the left side, while the canine relationship was undetectable because of the absence of the arch in the right upper canine and the contemporary presence of the permanent and the deciduous upper left canine. Therefore, the ectopic position was of a permanent one. A unilateral crossbite was present on the right side (Figure 7).

Overjet and overbite were increased; a severe dentoalveolar discrepancy with upper and lower crowding was evident. Oral hygiene was poor, with the presence of bacterial plaque, probably due to the heavily limited visual capacity. Decays were also present on elements 1.6, 6.5, 2.6, 3.6, and 3.7.

*(2) Radiographic Examination*. The orthopantomography of the dental arches revealed the presence of all the permanent teeth, the impacted upper right canine, persistence of the upper left deciduous canine, the second molar, the lower left deciduous second molar, and the eruptive delay of 3.5 with alteration of the axial inclination, mesial inclination of the lower second molars, and horizontal impaction under the crown of the first molars.

**Figure 8:**
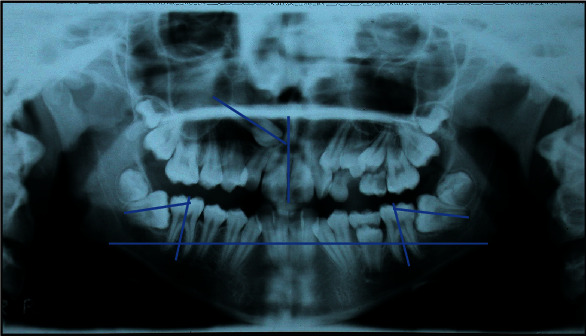
MZ L. P.'s panorex. The right canine *α* angle is 58°, and the vertical distance from the occlusal plane is 16 mm. The inclination of the second molar axis is 68° for 4.7 and 69° for 3.7.

The inclination of the second molar axis related to the first molar axis was 68° for 4.7 and 69° for 3.7 [12]. The inclination of the upper left canine was 58°, as evaluated with the *α* angle, and the vertical distance from the occlusal plane was 16 mm (Figure 8).

In this case, the presence of severe eruptive disturbances also suggested the prescription of a further radiographic evaluation with a TC Dentascan of the upper and lower arches.

**Figure 9:**
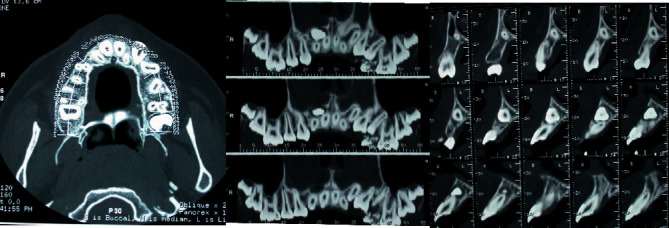
MZ L. P.'s upper arch Dentascan: upper right canine buccal impaction.

The X-ray observation revealed a high vestibular inclusion of 2.3. A tight contact was evident between the crown of the impacted canine and the apical third of the central and lateral left incisor roots. Slight signs of root resorption in the point of contact were advisable for the right lateral incisor. The crown of the upper left canine was superimposed on the gingival third of the lateral incisor root. An eruptive delay of 1.4 and 1.5 was detectable with a palatal axial inclination of 1.5 (Figure 9).

The evaluation of the two lower molars showed an H stage of maturity (Demirjian dental age assessment), acceptable for the age of the patient; the position of both the molars was horizontal with a tight contact with the lingual cortical bone.

**Figure 10:**
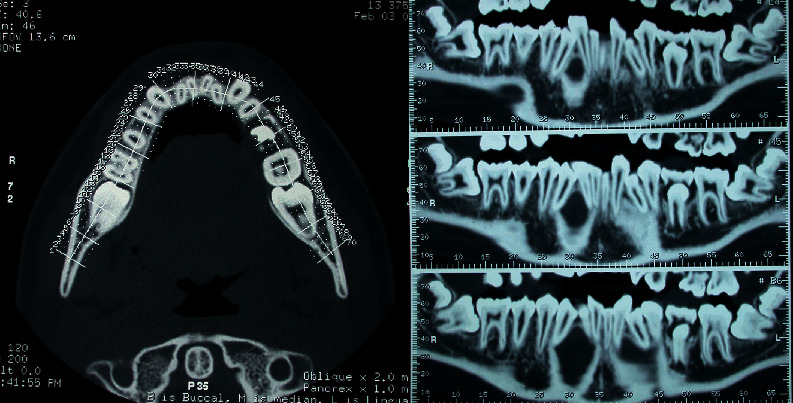
MZ L. P.'s lower arch Dentascan: deep mesial impaction of both the lower second molars.

Most of the occlusal surfaces of 4.7 and 3.7 were positioned under the crown equator of the respective first molar (Figure 10).

The cephalometric analysis of the latero-lateral radiography is summarised in Table 4. The results showed a skeletal class II malocclusion and an increase in the vertical dimension of the lower third. The maxillary and mandibular incisors were proclined.

### 1.6. Diagnostic Focus and Assessment

Both the twins showed a class I dental malocclusion with severe dentoalveolar discrepancy.

In the cast analysis of twin V. P., the lack of space was of -13 mm maxilla and -14 mm lower jaw (Table 1). In twin L. P., the discrepancy between the size of the teeth and those of the arches was equal to -11.5 mm in the upper arch and -13 mm in the lower arch (Table 3).

The latero-lateral radiography examination showed skeletal hyperdivergent class II but with signs of mandibular length and therefore a tendency of a class II skeletal pattern of growth; an excessive vestibular inclination of the upper and lower teeth was detected in both the cases.

A protrusive lower lip characterised the profile of both twins.

#### 1.6.1. Treatment Options

The study of clinical and radiographic data of the two sisters indicates the immediate need for orthodontic treatment, which must not be delayed in time (Index of Orthodontic Treatment Need- (IOTN-) 5) [16].

The most evident need of therapy is the presence of multiple inclusions, namely, for the upper canine, right in one sister and left in the other, and of both second molars in the lower arch for both sisters.

The prognosis of a possible surgical orthodontic approach for the impacted teeth, although some similarities were present in the sisters, was significantly different because of the localisation of the upper canine, vestibular or palatal, and the distance of the canine from the occlusal plane, which was far accentuated for the vestibular inclusion. The bilateral inclusion of the lower second molars showed a different degree of mesial inclination, more severe for L. P., in which the deep of the molars' crown was also heightened.

A serious dentoalveolar discrepancy was present in both the patients, leading to a consideration of a fixed multibracket treatment with extraction of four premolars; this is also acceptable for the presence of a protrusive profile.

A possible orthognathic surgery for the correction of the skeletal malocclusion was not considered because of the mild level of the discrepancy and global clinical, medical, and social consideration for which the family refuses to evaluate for this approach.

The treatment plan is therefore different for the two sisters.

For V. P., a surgical orthodontic approach was planned for upper left canine disimpaction, using a transpalatal arch with a cantilever spring soldered to the palatal bar [17]. The aim of the first step is to extrude the canine by a closed eruption technique. Only after the first radiographic evaluation of the effective movement of the canine can a subsequent fixed multibracket treatment, with extraction of four first premolars, take place. In the lower arch, the second molar disimpaction was considered with the aid of a De-Impactor spring (Dentamate Co.), positioned under the contact point between the first and second molars bilaterally [18]. Once free in the mesial part of the second molars, a complete bonding of the lower arch will take place.

For patient L. P., the surgical orthodontic approach was planned, considering the upper canine disimpaction, using the VISTA technique [19], which entails a closed subperiosteal traction, anchored to a transpalatal bar with a vertical arm vestibular to the upper first right molar. For this patient too, only after the first radiographic evaluation of the effective movement of the canine can a subsequent fixed multibracket treatment, with extraction of four first premolars, take place. The presence of an inclination of over 68° for both the second lower molars indicates the need for a different procedure for the recovery, using a cantilever spring, as described by Doshi et al. [20].

#### 1.6.2. Treatment Alternatives

Only if the canines are not responsive to the orthodontic traction, a possible surgical extraction will take place, planning the substitution of it with the first premolar and, therefore, treating the patients with three premolar extractions.

For L. P., a possible alternative treatment plan should be considered: the extraction of the second lower molars and the mesialization of the third molars.

For V. P., in case the orthodontic uprighting is not achieved, surgical repositioning of the second molars should be considered [21].

## 2. Discussion

Twin studies in medicine are an important source of information to understand the relative importance of the genetic or environmental origin of specific disorders. In monozygotic twins, the gene is equal to 100%, so if a character appears more frequently in monozygotic twins than the expected rate in siblings or dizygotic twins, the genetic factor is important. If not, it must consider greater environmental importance in the aetiology of problems found [22].

The case reported is peculiar under several aspects. A similar but specular canine impaction, both for the side and for the localisation of the tooth, is present in both monozygotic twins.

In the lower arch, however, the same alteration of the eruptive pattern is evident for the same teeth, although with a different degree of severity. The lower second molars showed the same direction of impaction, with mesial inclination, and the same clinical evidence of osteomucosal semi-inclusion.

**Figure 11:**
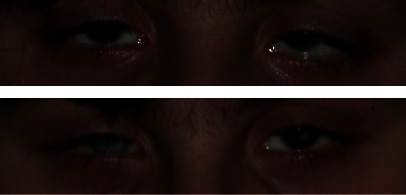
Detail of the clinical aspect of aniridia.

Moreover, the twins were also characterised for the presence of a congenital ocular malformation because the presence of aniridia was diagnosed early for both (Figure 11). Therefore, the case is interesting due to the simultaneous presence of occlusal and ocular abnormalities, especially in patients with a negative family history for both diseases.

Aniridia is defined as a rare clinical condition in which there is a partial or complete absence of the iris. The prevalence reaches 0.018% and is equally distributed among males and females. This condition is usually congenital; however, its aetiology is still uncertain. The congenital aniridia can be classified as autosomal dominant, autosomal recessive congenital, or sporadic congenital.

Sporadic aniridia can be detected between symptoms in certain clinical conditions, as it happens in WAGR syndrome, a rare genetic disorder characterised by Wilms' tumour, aniridia, genitourinary anomalies, and mental retardation, or in Gillespie syndrome, where it is present in the autosomal recessive congenital aniridia.

Aniridia can be associated with several other anomalies, including sensory deficiency such as abnormal olfaction or hearing loss, absent or hypoplastic patella, and the AGR triad (aniridia, genitourinary abnormalities, mental retardation) [23]. However, to the best of our knowledge, congenital aniridia has not been associated with dental anomalies.

While aniridia is a rare condition, the canine inclusion, particularly in the palatal side, is a frequent abnormality in the Caucasian population, being most common in females. If it is assumed that the prevalence of the upper canine inclusion is equal to 2% in the general population, the probability of finding the same palatal inclusion in monozygotic twins, as a sporadic event, would be questionable [24]. The frequent association of canine impaction with other dental anomalies, such as tooth number or size anomalies both in the same subject and in families, strongly suggests a genetic origin, and research progressively supports this hypothesis. It, therefore, seems likely that the inclusion is linked to genetic causes [25, 26].

The presence of palatal inclusion of the canine in monozygotic twins has already been reported in the literature. Leonardi et al. [6] report the case of monozygotic twins with palatal inclusion of the canine but in a specular position. In the cases described by the authors, the two lateral incisors were of normal size. From the study of these cases, the authors derive evidence to support the genetic aetiology of palatal inclusion of canine, rather an aetiology connected to the lack of guidance for lateral incisor anomaly [27, 28]. In this study, the twins also showed abnormalities of the position of the lower second molars, showing a mesial inclination. The presence of multiple abnormalities in the same subjects has been variously reported in the literature; the concomitant presence of the most various anomalies, including alterations of the eruptive pattern of the molars, was considered further proof of the genetic cause of these alterations.

In our reported cases, both patients presented with the inclusion of the upper canine and retention of the lower second molars, which showed an altered mesial inclination. This then presents great similarity with Leonardi et al.'s study, but the inclusion is specular not only in the side but also at the position, which is present in one patient in a palatal position and in a buccal position in another one. Moreover, in both patients, there was a considerable dentoalveolar discrepancy with significant space deficit.

While the literature has found significant evidence of the genetic aetiology of palatal canine inclusion, only a few studies report an association between dental anomalies and buccal inclusion [29]. Recently, some authors developed a different theory to explain the possible aetiology of both canine impactions from the vestibular side and on the palatal side: according to this theory, both the canines included in the palatal and buccal position have a genetic origin, but local factors can lead to displacement in the palatal or vestibular direction [30].

The “sequential theory,” developed on the basis of a large retrospective study, affirms that genetic and environmental factors act at different times, and therefore, both the palatal and buccal impacted canines have an initial genetic cause while the specific clinical picture is realised according to environmental influences, predominantly referred to as excess of space, as it happens in microdontia. However, previous studies still consider the lack of space a possible local cause for vestibular inclusion. In the monozygotic twins, the lack of space was evident.

The detection of the presence, however, is of very similar alterations in both patients, which shows a correlation in the elements involved in abnormalities—upper canines and lower second molars—and the correlation of dental size detected and the presence of similar values of dentoalveolar discrepancy, which seems to indicate a genetic basis for these characteristics.

The observation, in the same subjects, of ocular congenital anomalies is not easy to analyse, given that research has only recently identified several genes involved in the pathogenesis of aniridia, such as the regulatory region deletions in PAX6 and PITX2 and, in cases of anterior segment dysgenesis (ASD) disorders, mutations in the alpha-1 collagen type IV (COL4A1) and beta-1,3-galactosyltransferase-like (B3GALTL) [11, 31, 32].

These conditions are not, however, related to concurrent dental alterations.

In the genesis of ocular development, alterations then reconnect with genetic alterations in which the transcription factors have an important role, in particular some DNA-binding transcription factors such as PAX6, FOXC1, SOX2, FOXE3, OTX2, PITX2, and PAX2 [33].

Alterations in the same PITX2, FOXC1, and PAX6 genes, linked to important dental abnormalities, were identified in other clinical conditions such as Rieger syndrome [34, 35].

Therefore, only on a speculative level, it could be imagined as an alteration, intervened in the gestational period and capable of inducing alterations in the same embryological derivation areas.

In the cases presented, the parents did not refer to genetic analysis at birth or in the moment of congenital aniridia diagnosis. A possible deepening of the diagnosis of the underlying causes of aniridia was refused by the parents in that moment because the ocular pathology treatment possibilities were not influenced from a more accurate genetic knowledge and for economic reasons. Alike, the parents refused the genetic analysis also when the multiple dental impaction became evident.

Some clinical considerations influenced this decision; several conditions involving the presence of multiple impactions are known to be related to a genetic mutation that also involves the failure of all the orthodontic strategies of recovery of the impacted teeth. Nevertheless, some clinical observations can lead to substantially excluding these conditions for the MZ, as it happens for the primary failure of eruption (PFE). In the PFE cases, in fact, the teeth involved are mostly located on only one side, in the posterior area, and showing a progressive worsening of the impaction from the first tooth to the last one of the areas affected. This condition is clinically evident with a posterior open bite. Similarly, a differential diagnosis is possible on a clinical base also for the anchyloses, clinically characterised for a single tooth involved and with a vertical alteration of its position [36]. The treatment choices in the other cases of impaction always comprehend a treatment with an orthodontic-surgical approach.

For the MZ, therefore, a deepening of the diagnostic procedures adding a genetic mapping would not change in the treatment plane and was therefore refused by the patients and their parents.

However, not having subjected our patients to a genetic investigation, the data of the present study is limited to a simple hypothesis that suggests a possible diagnostic genetic study in cases with similar dental and ocular abnormalities.

## 3. Conclusions

The debate about the aetiology of inclusions sees many points that are still under discussion, especially the different genetic bases of the inclusions in the front or rear area or different importance of the genetic factor in the inclusion of the upper canines. Finally, the possibility that alterations in the path and of the dental element eruptive potential may be associated with other congenital problems broadens the range of possibilities of aetiological causes of the inclusions.

The present case study of the monozygotic twins provides information on the possible genetic basis of dental inclusions. The potential link between inclusions and abnormalities of eruption and other genetic alterations can push towards finding genetic factors involved in the anomalies. This then affects both the possibilities of early diagnosis of clinical conditions that require treatment with chances of therapy success. It particularly affects the determination of the aetiology of malocclusion's tractability. The possible genetic aetiology can improve the chances of early interception but does not allow preventive treatments solely based on changing environmental conditions.

## Figures and Tables

**Table 1:** V. P. twin dental formulae (^∗^semi-inclusion). Monozygotic twin V. P.'s dental formula as in ISO (International Organization for Standardization) 5 with the mesiodistal (MD) crown diameters of permanent teeth (in mm to the nearest 0.5 mm). Bold numbers report the mesiodistal dimension of a deciduous tooth, and italic numbers report the mesiodistal dimension of an unerupted tooth, evaluated on the panorex and calculated based on the magnification coefficient.

Twin V. P.: tooth number (ISO two-digit system)														
Maxillary teeth														
Upper arch		16	15	14	13	12	11	21	22	63	24	25	26	
MD diameter (mm)		11	8	7.5	8	7	9	9	7	**6** *8*	7.5	8	11	
Dentoalveolar discrepancy = −13 mm														

Mandibular teeth														
Lower arch	47^∗^	46	45	44	43	42	41	31	32	33	34	75	36	37^∗^
MD diameter (mm)		11	8	7.5	7.5	6.5	6	6	6.5	7.5	7.5	**9.5** *8*	11	
Dentoalveolar discrepancy = −14 mm														

**Table 2:** MZ V. P.'s cephalometric analysis.

MZ V. P.	
Maxilla to cranial base	
SNA (°)	80.4
Mandible to cranial base	
SNB (°)	76.2
SN-GoGn (°)	45.2
FMA (MP-FH) (°)	32.1
Maxillo-mandibular	
ANB (°)	4.2
Maxillary dentition	
U1-NA (mm)	4.7
U1-SN (°)	103.1
Mandibular dentition	
L1-NB (mm)	9.2
L1-MP (°)	88.6
Soft tissue	
Lower lip to E-plane (mm)	0.9
Upper lip to E-plane (mm)	-10.5

**Table 3:** L. P. twin dental formulae (^∗^semi-inclusion). Monozygotic twin L. P.'s dental formula as in ISO (International Organization for Standardization) 5 with the mesiodistal (MD) crown diameters of permanent teeth (in mm to the nearest 0.5 mm). Bold numbers report the mesiodistal dimension of a deciduous tooth, and italic numbers report the mesiodistal dimension of an unerupted tooth, evaluated on the panorex and calculated based on the magnification coefficient.

Twin L. P.: tooth number (ISO two-digit system)														
Maxillary teeth														
Upper arch		16	15	14	/	12	11	21	22	23/63	/	65	26	
MD diameter (mm)		11	7.5	7.5	8	7	9	9	7	**8.5** *6*	8.5	**9** *7.5*	11	
Dentoalveolar discrepancy = −11.5 mm														

Mandibular teeth														
Lower arch	47^∗^	46	45	44	43	42	41	31	32	33	34	75	36	37^∗^
MD diameter (mm)		11	8	7.5	7.5	6.5	6	6	6.5	7.5	7.5	**9.5** *8*	11	
Dentoalveolar discrepancy = −13 mm														

**Table 4:** MZ L. P.'s cephalometric analysis.

MZ L. P.	
Maxilla to cranial base	
SNA (°)	83.1
Mandible to cranial base	
SNB (°)	78.4
SN-GoGn (°)	41.3
FMA (MP-FH) (°)	28.9
Maxillo-mandibular	
ANB (°)	4.7
Maxillary dentition	
U1-NA (mm)	6.5
U1-SN (°)	106.9
Mandibular dentition	
L1-NB (mm)	13.9
L1-MP (°)	93.2
Soft tissue	
Lower lip to E-plane (mm)	-0.5
Upper lip to E-plane (mm)	-9.0

## Additional Points

*Guidelines*. The authors used appropriate guidelines (CARE for case reports).
